# CMOS-fabricated dielectrophoretic chip with embedded 3D TiN nano-electrode arrays for sperm capture and sperm damage reduction

**DOI:** 10.3389/fbioe.2025.1635799

**Published:** 2025-07-14

**Authors:** I-Hsuan Liao, Jeng-Huei Shiau, Chao-Min Cheng

**Affiliations:** ^1^ Institute of Biomedical Engineering, National Tsing Hua University, Hsinchu, Taiwan; ^2^ NEAT Biotech Inc., Hsinchu, Taiwan

**Keywords:** dielectrophoresis, embedded 3D TiN nano-electrode arrays, CMOS-fabricated, boar sperm, bovine sperm

## Abstract

**Introduction:**

This study developed an embedded 3D TiN nano-electrode arrays with a strong electric field and high biocompatibility for the effective capture of motile sperms. The chip integrates CMOS fabrication technology with a three-dimensional structural design, exhibiting both electrode-based dielectrophoresis and insulator-based dielectrophoresis characteristics. It provides a stable operating environment under strong electric fields with minimal Joule heating interference.

**Methods:**

In the experiments, we evaluated the effects of different waveforms and capture spaces on the capture efficiency of boar and bovine sperms.

**Results and discussion:**

The results showed that square waves improved capture efficiency by approximately10% and confirmed that the capture space must exceed half the sperm length to enhance efficiency. When operated under a 20 Vpp square wave, the chip only generated a temperature rise of 1.7°C, causing no significant damage to the sperms and no notable decrease in viability. Under optimal conditions, the capture efficiencies for boar and bovine sperms reached 65.54% ± 1.07% and 63.25%, respectively. Overall, the results demonstrate that this chip offers high throughput, low Joule heating interference, and good species adaptability, showing potential for use in dynamic cell capture and high-throughput analysis.

## Introduction

Dielectrophoretic (DEP) force has been extensively studied for particle manipulation and separation, demonstrating effective enrichment and separation performance in both biological and non-biological samples (Morgan et al., 1999; Pesch and Du, 2021). However, due to limitations in device design, Joule heating not only reduces the effectiveness of the dielectrophoretic force but also poses a significant challenge when applied to biological samples. This issue is particularly critical for temperature-sensitive sperm samples, as excessive Joule heating can rapidly reduce their motility or even lead to cell death. Since sperm motility is one of the key indicators of sperm quality (Chakraborty and Saha, 2022), maintaining it is crucial. However, highly motile sperms, due to their strong locomotion ability, are also more likely to escape the influence of the dielectrophoretic force. As a result, the application of dielectrophoresis in sperm manipulation has been relatively underexplored in literature, with few studies directly addressing sperm capture efficiency. In our study, we utilized embedded 3D TiN nano-electrode arrays to capture sperms, and through innovative structural design, we successfully captured highly motile sperms without compromising their viability.

With the advancement of technology, conventional electrode-based dielectrophoresis (eDEP) devices, which are prone to generating Joule heating, have gradually evolved into insulator-based dielectrophoresis (iDEP) systems that offer higher biocompatibility. In electrode-based dielectrophoresis, a non-uniform electric field is generated by electrodes to produce dielectrophoretic force. Due to fabrication constraints, metal thin layer deposited by thin-film process are commonly used as electrodes, typically with dimensions on the micrometer scale. The electric field is concentrated at the sharp tips of the electrodes. To effectively enhance both the electric field strength and the effective region, various electrode geometries have been designed, such as grid-like crossed planar electrodes (Abd Rahman et al., 2017; Zhang et al., 2019). Although electrode-based dielectrophoresis provides stronger electric fields, the large contact area between the electrodes and highly conductive biological sample solutions often leads to significant Joule heating. Prolonged exposure can damage biological samples and reduce the effectiveness of the dielectrophoretic force. To mitigate these negative effects, many electrode-based dielectrophoresis devices adjust electrode geometry to enhance field strength and thus reduce the required voltage.

In response to these challenges, insulator-based dielectrophoresis technology was introduced. It features a layer of insulation that separates the metal electrodes from the sample solution, preventing direct contact and thereby minimizing Joule heating. The principle of insulator-based dielectrophoresis involves inserting insulating structures into microchannels, which compress the electric field lines and create variations in field density, thus forming a non-uniform electric field (Abd Rahman et al., 2017). However, due to the insulating barrier, insulator-based dielectrophoresis typically requires several hundred to several thousand volts to generate sufficient dielectrophoretic force—several times higher than the tens of volts typically used in electrode-based dielectrophoresis (Julius et al., 2023). Furthermore, due to design limitations, both electrode-based dielectrophoresis and insulator-based dielectrophoresis generally require integration with microfluidic channels for fluid control, limiting their throughput to less than 1 mL/h (Pesch and Du, 2021; Lien and Yuan, 2019).

In this study, we utilized embedded 3D TiN nano-electrode arrays fabricated using CMOS processing technology for dielectrophoretic capture of sperms. CMOS fabrication technology overcomes the feature size limitation of conventional dielectrophoretic chips. Dielectrophoretic chips fabricated using the MEMS process typically rely on standard BEOL metal patterning, which is generally limited to the microscale feature size (Lu et al., 2024). In contrast, CMOS fabrication technology enables the fabrication of nanoscale electrodes, allowing our chip to generate a stronger electric field and expanding the effective region for the dielectrophoretic-based manipulation. By reducing the electrode dimensions to the nanoscale, the electric field strength is enhanced by approximately fivefold compared to conventional microscale electrodes (Lien and Yuan, 2019), while significantly minimizing the contact area with highly conductive solutions, thereby reducing Joule heating. The chip is designed with an insulating layer that isolates the heat-generating metal lines, and an elevated insulating structure is built around the electrodes to embed them in a bowl-shaped recessed configuration. This non-contact electrode structure retains the key characteristics of insulator-based dielectrophoresis by reducing both Joule heat conduction to the chip surface and direct contact between the electrodes and sperms, demonstrating the improved biocompatibility compared to the chip used in our previous studies (Lu et al., 2024; Liao et al., 2025). The relative position of the sperm and the electrodes is illustrated in Supplementary Figure S1. This structure design enables a stronger electric field than conventional electrode-based dielectrophoresis as well, thereby enhancing the sperm capture efficiency. With its scalable design, the chip effectively improves processing efficiency. In addition to reducing Joule heating interference and increasing throughput, the chip also features adjustable electrode spacing, allowing the dielectrophoretic capture conditions to be optimized based on the sperm size of different species. This capability was verified in our preliminary study using boar sperms (Liao et al., 2025).

**FIGURE 1:**
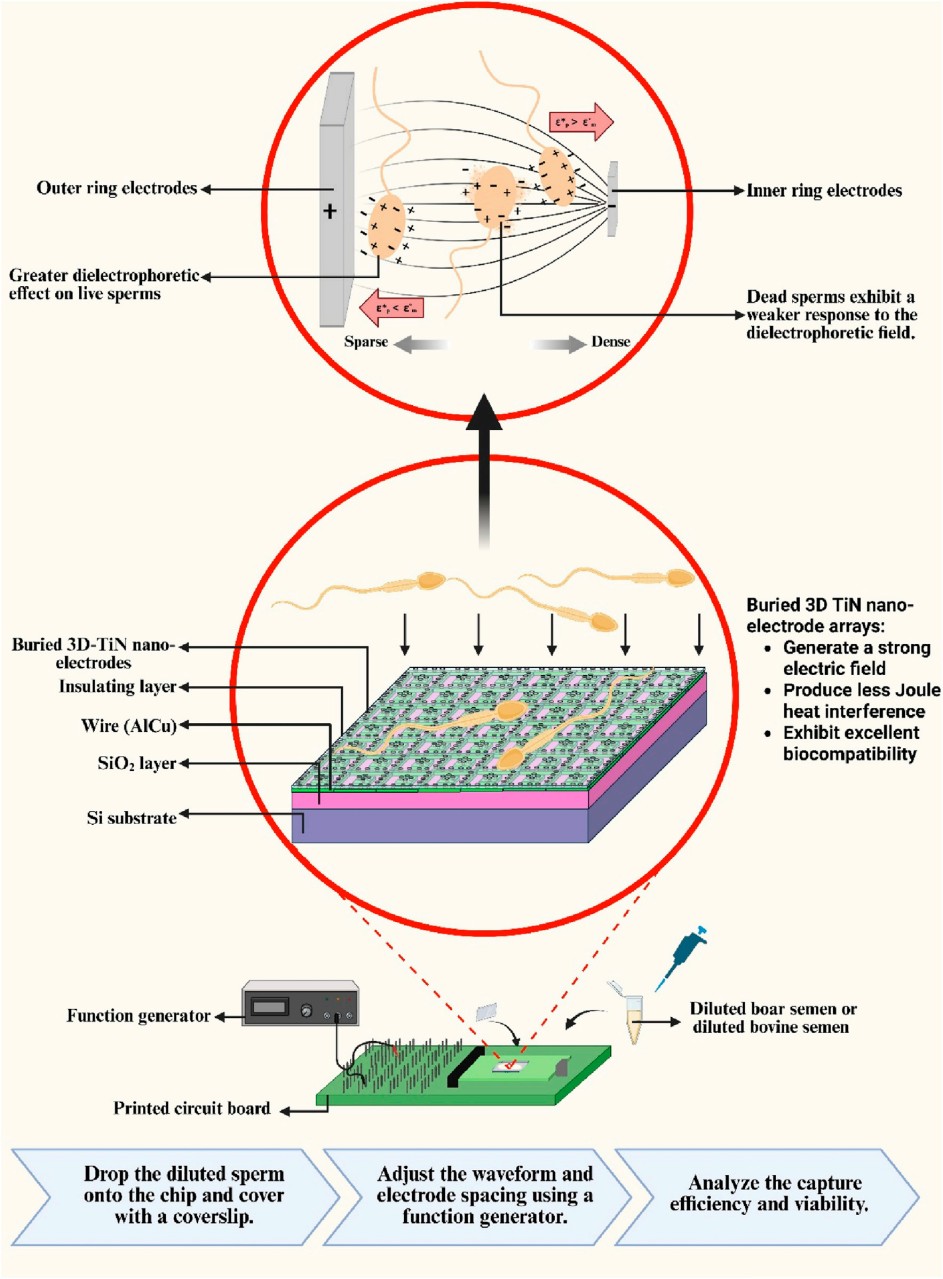
Schematic of the sperm capture method.

In previous experiments, we demonstrated that motile sperms can be effectively captured by dielectrophoretic force due to their superior polarizability. We also investigated the effects of operating parameters such as applied voltage, applied frequency, and electrode spacing on sperm capture efficiency (Lu et al., 2024; Liao et al., 2025). In this study, we further explore the influence of waveform on sperm capture performance. According to the literature, waveform changes affect the effective root-mean-square voltage (Vrms); for example, sine and square waves differ by a factor of $\sqrt{2}$ , which in turn alters the strength of the dielectrophoretic force (Boldt et al., 2025). Due to concerns about Joule heating, square waves have rarely been applied in sperm dielectrophoresis studies. However, this study overcomes such limitations, enabling the investigation of waveform optimization for improved capture efficiency. Finally, we demonstrate the chip’s adaptability and capture performance for different species—bovine and boar sperms—by adjusting the electrode spacing accordingly. Figure 1 illustrates the capture method and experimental concept adopted in this study. Therefore, this study aims to investigate the dielectrophoretic capture performance of an embedded 3D TiN nano-electrode arrays, which offers both high electric field strength and excellent biocompatibility, under different waveform conditions. The capture efficiency and viability of sperms from different species are evaluated to assess the chip’s biological compatibility. By adjusting operating parameters and electrode structure, this study seeks to optimize sperm capture conditions and further validate the feasibility and potential of this chip for high-throughput and biocompatible sperm manipulation applications.

## Materials and methods

### Fabrication of embedded 3D TiN nano-electrode arrays

**FIGURE 2:**
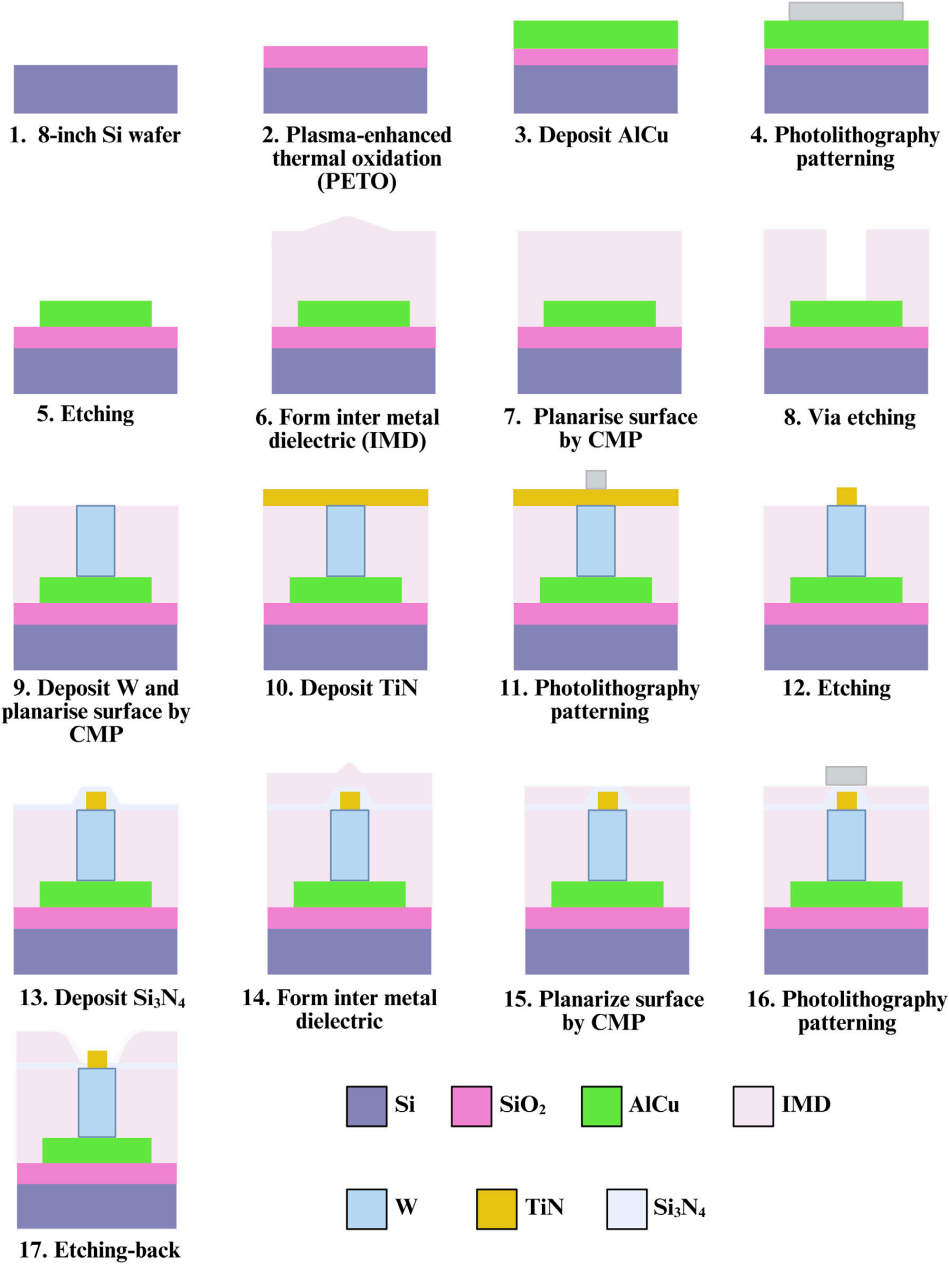
Chip fabrication flowchart.

**FIGURE 3:**
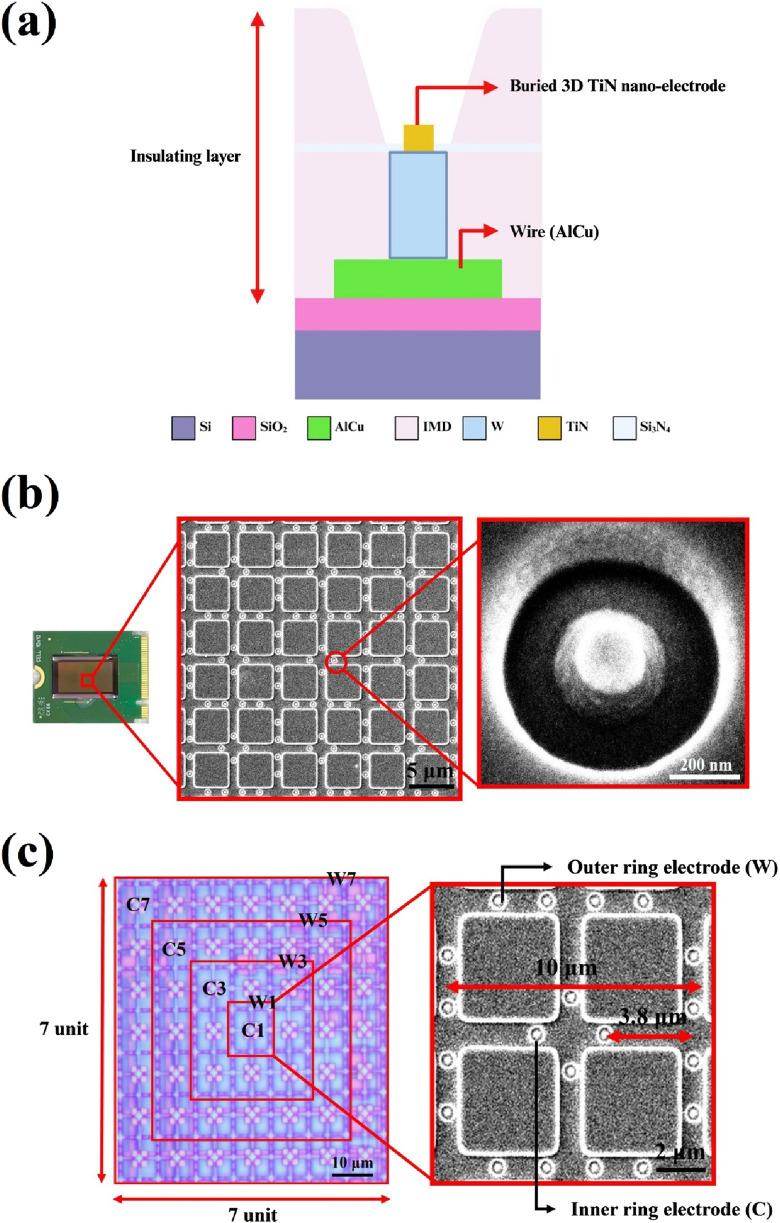
Structure and control unit of embedded 3D TiN nano-electrode arrays. **(a)** Schematic of the cross section of single electrode, **(b)** embedded 3D TiN nano-electrode array SEM image and **(c)** electrode control unit.

The primary design goal of this chip is to minimize potential damage to sperm during dielectrophoretic capture. To achieve this, an elevated insulating layer was fabricated around the electrodes, embedding them at the bottom of a bowl-shaped recessed area to form a non-contact electrode structure. For the fabrication process, steps 1 through 15 were adapted from a previous study (Liao et al., 2025), with a key modification: an additional photolithography and development step was introduced prior to the etching process to mark the electrode regions. The insulating layer in these designated areas was then removed using a etching-back process to form the required electrode structures (steps 16–17). The complete fabrication process is illustrated in Figure 2. Due to the nature of the process, the resulting electrodes resemble a pudding-like shape, featuring a smoothly rounded top with a diameter of about 0.2 μm that transitions into a wider conical base, forming non-contact nano-electrodes. A schematic cross-sectional view of single 3D TiN nano-electrode is shown in Figure 3a. Although single electrode does not form a cylindrical shape as in our previous studies (Lu et al., 2024; Liao et al., 2025) due to the fabrication constraints, the diameter at the top of the electrode remains the same. Therefore, the electric field strength generated by the electrode would be not significantly different from that with previous designs (Lien and Yuan, 2019; Lu et al., 2024; Liao et al., 2025).

### Electrode structure and arrays of the embedded 3D TiN nano-electrode arrays

Figure 3b presents scanning electron microscope (SEM) images of the electrode arrays and a single electrode on the chip. The electrodes are arranged in a square grid, and the surrounding elevated insulating layer is clearly visible. This design effectively increases the distance between the biological sample and heat-generating components on the chip (such as wires and electrodes), thereby reducing the impact of Joule heating on the sample.

Another key feature of the chip is its adjustable electrode spacing, which allows the distance between the inner and outer ring electrodes to be tuned according to the sperm size of different species. Figure 3c shows the design of the electrode arrays. The left panel displays the view of one control unit under an optical microscope, while the right panel shows an SEM image of a basic working unit. As shown, each control unit is a square consisting of 7 × 7 basic units. Each basic unit comprises an inner ring of four electrodes and an outer ring of sixteen electrodes. The difference in the number of electrodes between the inner and outer rings creates a non-uniform electric field. Each basic unit has a square side length of 10 μm, and the distance between the inner ring and outer ring electrodes is 3.8 μm as shown in Figure 3c. The electrode control scheme follows our previous design (Liao et al., 2025). Each control unit can selectively activate four central (C) electrodes and four window (W) electrodes, namely, inner ring electrodes C1, C3, C5, and C7, and outer ring electrodes W1, W3, W5, and W7. Different electrode combinations generate varying sperm capture spacings, thus influencing capture efficiency and performance. For example, the spacing between electrodes C1 and W1 is 3.8 μm, while that between C1 and W3 is 13.8 μm, and so on. In our previous study, we defined the corresponding sperm capture space, referred to as the effective dielectrophoretic region, for each electrode spaceing (Liao et al., 2025). For instance, the C1+W1 combination results in a 10 μm capture space, while the C1+W3 combination yields a 30 μm capture space.

### Sperm preparation

Fresh semen from Landrace boars and frozen semen from Holstein bovines were used in this study. Boar semen samples were obtained from the Animal Technology Research Institute (Miaoli, Taiwan), while frozen bovine semen was provided by Chien Ying Company LTD. (Taipei, Taiwan).

Higher conductivity not only reduces the strength of the dielectrophoretic force but also leads to increase Joule heating during the dielectrophoretic operation, which could increase the solution temperature (Park et al., 2020; Tada and Seki, 2022). Therefore, we used a low-conductivity dielectrophoresis buffer to dilute the semen samples. To minimize the variation between each experiment, all samples were processed and diluted to achieve a conductivity range of 500–900 μS/cm. This specific range was selected to ensure sufficient dielectrophoretic force (Park et al., 2020) while maintaining an adequate number of sperms within the field of view, attempting to reduce the observation variation. The dielectrophoresis buffer was prepared based on a formulation reported in the literature (Park et al., 2020), and the conductivity was measured using a Bante530 portable conductivity meter (Sugar Land, United States).

Boar semen was incubated in a 37°C water bath for 20 min before assessment. Since sperm quality could be influenced by weather, transportation, and individual differences, both motility and concentration were evaluated using the iSperm system (Aidmics Biotechnology, Taipei, Taiwan). Samples were excluded once the sperm motility was below 70%, the sperm concentration was too low, or the severe sperm agglutination was observed. Qualified samples were gradually diluted with dielectrophoresis buffer until the conductivity reached the target range. Frozen bovine semen was thawed in a 37°C water bath for 30 s. After thawing, both motility and concentration were also assessed using the iSperm system. The qualified samples were then diluted with dielectrophoresis buffer to adjust its conductivity within the specified range.

Some studies have employed differences in certain characteristics between X and Y sperms for sex selection purposes (Katigbak et al., 2019), and others have investigated their differential responses to dielectrophoretic forces (Wongtawan et al., 2020; Dararatana et al., 2015; Koh and Marcos, 2014). However, in this study, to ensure comparable experimental conditions between boar and bovine sperms, we used mixed-sex sperm samples without sex sorting and did not perform capture analysis targeting a specific sperm sex.

### Sperm capture via dielectrophoresis

The cleaned chip was mounted on a printed circuit board (PCB), and wires were connected to a waveform generator (MFG-2260MFA, Gwinstek, New Taipei, Taiwan), which allowed adjustment of applied frequency and voltage. Then, 20 μL of diluted semen was dropped onto the chip. After covering it with a coverslip, observations and recordings were performed using a metallurgical microscope (M835, MICROTECH, Shang Chen Optical International Co., Ltd., Taipei, Taiwan).

The total recording time was 2 min: the first 30 s captured the natural behavior of the sperms, followed by 30 s of dielectrophoretic capture with the waveform generator turned on. Finally, the generator was turned off, and sperm behavior was recorded for another 1 min to observe changes after the dielectrophoretic effect ended. The video allowed comparison of sperm behavior before and after dielectrophoretic application. Video analysis was conducted using the MicroCamV8 software provided by Shang Chen Optical International Co., Ltd. (Taipei, Taiwan). The total number of motile sperms in the field of view before dielectrophoretic activation and the number of sperm lingering or rotating within the active region after dielectrophoretic activation were counted. These values were used in Formula 1 to calculate the sperm capture efficiency.

$$\text{Dielectrophoretic capture efficiency of sperm }\left(\%\right)=\frac{\left(\text{Number}\;\text{of}\;\text{captured}\;\text{sperms}\;\text{in}\;\text{dielectrophoretic}\;\text{buffer}\right)}{\left(\text{Total}\;\text{live}\;\text{sperms}\;\text{in}\;\text{dielectrophoretic}\;\text{buffer}\right)}\times 100\%$$ (1)

### Sperm viability

To evaluate the impact of dielectrophoretic capture on sperm viability, 20 μL of diluted semen was dropped onto the chip and covered with a coverslip. Two testing conditions were applied: one without dielectrophoretic force and the other with an applied 20 Vpp dielectrophoretic force applied for 30 s. Following testing, dead sperm staining was performed to assess whether sperm viability was affected. Sperm staining analysis was conducted using Eosin Y-Nigrosin solution (Baso Biotech, Taipei, Taiwan). The test sample was mixed thoroughly with the staining solution at a 1:1 volume ratio and allowed to sit for 30 s. Then, 5 μL of the mixture was dropped onto a glass slide to prepare a smear, which was air-dried before observation. Under an optical microscope, 200 sperms were randomly observed in each field of view. The numbers of stained and unstained sperms were counted to evaluate sperm viability (Mortimer, 2020). Each experimental condition was repeated five times to ensure result reliability.

### Joule heating calculation of electrodes and measurement of chip temperature rise

We employed cyclic voltammetry (CV) to measure the current generated by the chip during operation. The measured current and an applied voltage of 10 V were substituted into Formula 2 to calculate the accumulated Joule heat after 30 s of operation, under the assumption that the phase difference between voltage and current (∅) is zero:

$$P=V_{\text{rms}}\times I_{\text{rms}}\times \cos\emptyset$$ (2)

In addition, an infrared thermometer was used to measure the temperature change of the chip before and after operation. Under the conditions of square wave input, applied frequency 3 MHz, applied voltage 20 Vpp, and full electrode activation, temperature measurements were conducted for durations of 30 s, 1 min, and 2 min. Each condition was repeated three times to obtain the average temperature difference.

### Data analysis

Statistical analysis was performed using GraphPad Prism version 8.4 (GraphPad Software, CA, United States). The comparison of sperm viability between two groups was assessed using Student’s t-test, and results were presented as box plots. A p-value of less than 0.05 was considered statistically significant.

For the analysis of capture efficiency, each experimental condition was repeated five times, and differences among groups were evaluated using one-way ANOVA. A p-value less than 0.05 indicated statistically significant differences among the conditions. Due to the concentration differences between fresh boar semen and frozen bovine semen, and the need to maintain conductivity within the 500–900 μS/cm range, the number of sperm observed under the field of view varied between the two sample types. Approximately 200–300 sperms were counted per experiment for boar semen, whereas only about 20–40 sperms were observed per experiment for frozen bovine semen. This difference in sperm count resulted in varying error bar sizes between the two datasets. Supplementary Table S1 summarizes the differences in conductivity between boar and bovine semen, as well as the number of sperm observed.

## Results and discussion

### Joule heating calculation and temperature rise measurement

The measured current generated by the chip electrodes under a 10 V input was 5.16 × 10^−8^ A. Based on this, the Joule heat generated per second in the 7 × 14 mm area was calculated to be 5.16 × 10^−7^ W, resulting in a total accumulated heat of 1.55 × 10^−5^ J over the 30-s duration of the dielectrophoretic operation. In addition to this calculation, we experimentally measured the temperature changes of the chip before and after dielectrophoretic operation. The results are summarized in Table 1. Using a square wave signal, fully activated electrodes, applied frequency of 3 MHz, and applied voltage of 20 Vpp, we measured the temperature rise after 30 s, 1 min, and 2 min of operation. The results showed minimal temperature differences. Compared with other dielectrophoretic systems under the same volume condition (50 μL), the temperature increase after a 30-s dielectrophoretic operation with applied 20 Vpp square wave using our 3D TiN nano-electrode arrays was only 1.70°C—lower than the 3.3°C reported in previous studies (Tada and Seki, 2022), demonstrating the superior performance of our chip in minimizing Joule heating effects.

**TABLE 1:** Measured temperature difference (n = 3).

Capture time	Temperature difference
30 s	1.7
1 min	4.2
2 min	5.4

Moreover, the sperm capture experiments were not conducted under the full-electrode activation condition, indicating that actual temperature rises during operation were even lower. According to the literature, elevated temperatures can lead to increased reactive oxygen species (ROS) generation in sperms, and prolonged exposure to temperatures above 40°C can reduce sperm motility and viability due to energy depletion and excessive ROS (Li et al., 2023). In our study, each dielectrophoretic capture experiment lasted only 30 s, and the temperature increase was consistently below 1.7°C, suggesting that the thermal damage to sperms was minimal.

### Sperm viability

**FIGURE 4:**
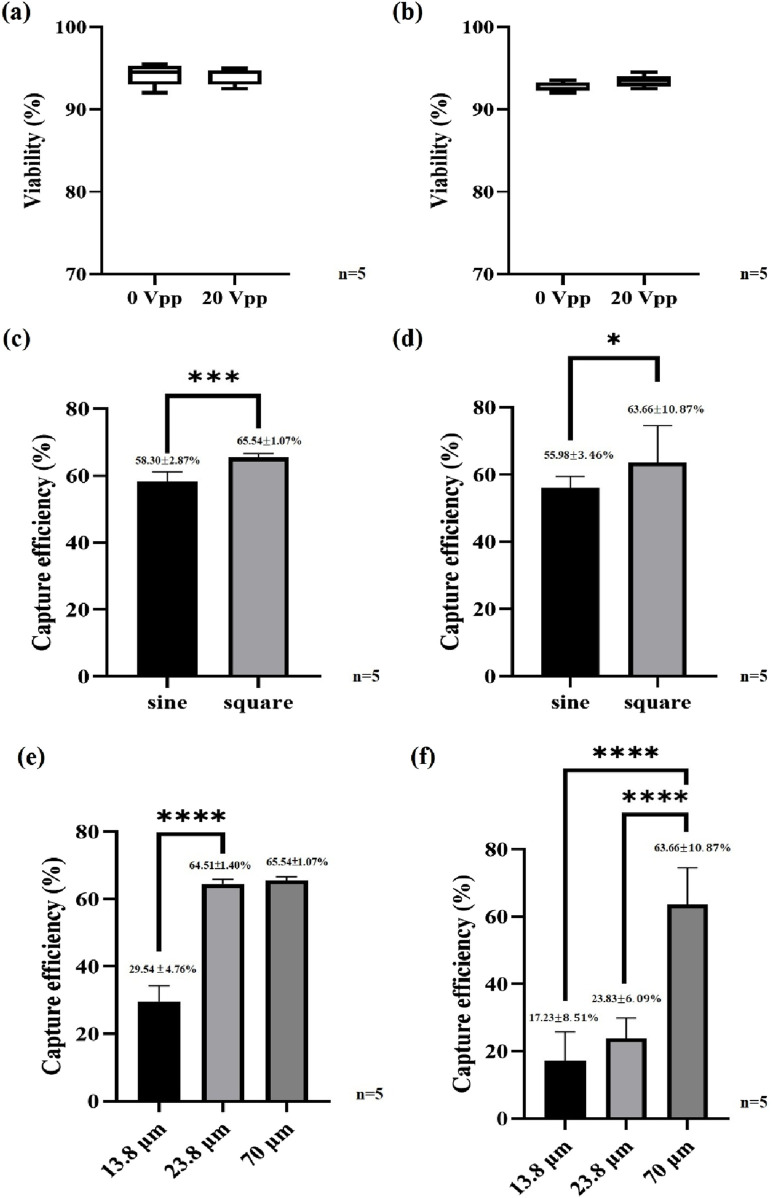
Sperm viability and capture efficiency. **(a)** Boar sperm viability, **(b)** bovine sperm viability, **(c)** effect of the waveform on the boar sperm capture efficiency, **(d)** effect of the waveform on the bovine sperm capture efficiency, **(e)** effect of the electrode spacing on the boar sperm capture efficiency and **(f)** effect of the electrode spacing on the bovine sperm capture efficiency. ***P < 0.001 and ****P < 0.0001.

The most prominent feature of the embedded 3D TiN nano-electrode arrays lies in its non-contact electrode design. This structure not only significantly reduces the contact area between the sample and the electrodes but also minimizes Joule heating interference through the use of a thickened insulating layer. As a result, potential damage to sperms during dielectrophoretic manipulation is greatly reduced. To verify the impact of this design on sperm viability, we assessed the viability of boar and bovine sperms before and after dielectrophoretic capture. The experiments were conducted under optimized capture conditions, including square wave signals, applied voltage of 20 Vpp, applied frequency of 3 MHz, and the C1+W7 electrode configuration. Figures 4a, b show the comparison of sperm viability for boar and bovine sperms, respectively, before and after dielectrophoretic capture. The results indicated no significant difference in viability for either species, suggesting that the chip’s effect on sperms under these operating conditions was minimal. This confirms that the combination of the thickened insulating layer and the non-contact electrode structure effectively reduces potential damage to sperms and enhances the biocompatibility of the overall operation.

### Effect of waveform on sperm capture efficiency

According to previous studies, using square waves as the electric field source can effectively enhance the efficiency of dielectrophoretic manipulation. At the same amplitude, the root mean square voltage (Vrms) of a square wave is $\sqrt{2}$ times that of a sine wave, generating a stronger electric field and, consequently, a larger dielectrophoretic force on the particles, thereby improving capture efficiency (Boldt et al., 2025; Contreras Dávila et al., 2016). However, in earlier dielectrophoresis-related studies, sine waves were commonly used instead of square waves because the devices tended to generate considerable Joule heating during operation. To avoid potential thermal damage to temperature-sensitive sperms, researchers opted for sine wave signals to minimize thermal effects (Wongtawan et al., 2020; Dararatana et al., 2015; Shuchat et al., 2019). In contrast, the embedded 3D TiN nano-electrode arrays used in this study feature a non-contact electrode design and a thickened insulating layer, which effectively isolates biological samples from Joule heat. This allows the use of higher electric field strength through square waves for sperm capture under low thermal interference and low-damage conditions. This provides a new feasible strategy to enhance capture efficiency without compromising biocompatibility.

To investigate the effect of waveform on dielectrophoretic capture efficiency, we conducted experiments comparing sine and square waves under the same conditions: applied frequency of 3 MHz, applied voltage of 20 Vpp, and the C1+W7 electrode configuration. Figure 4c presents the boar sperm capture efficiency under different waveforms. The results clearly showed that square waves significantly enhance sperm capture efficiency. Under the specified conditions, the capture efficiency using square waves reached 65.54% ± 1.07%, approximately 10% higher than that achieved with sine waves. This result highlighted the significant impact of waveform on dielectrophoretic efficiency and confirmed the advantage of square waves in generating stronger electric fields for improved sperm capture performance. Similarly, in bovine sperm capture experiments, we observed results consistent with those from boar sperms, as shown in Figure 4d. The results demonstrated that square waves also outperformed sine waves in capturing bovine sperms. Notably, the chip exhibited excellent capture performance for sperms from both boars and bovines, indicating its promising cross-species applicability and potential for broader biological applications.

### Effect of capture space on sperm capture efficiency across different species

In our previous study, we defined the sperm capture space corresponding to different electrode spacings and pointed out that, to effectively trap sperms, it is necessary to immobilize the sperm head and at least half of the tail. In other words, the capture space should be at least half the sperm length to achieve efficient trapping (Liao et al., 2025). Building on this concept, the present study further investigates how electrode spacing (i.e., the size of the capture space) affects the capture efficiency of sperms with varying lengths, using different species as experimental models. The selected Landrace boar sperms are approximately 50 μm in total length (Szablicka et al., 2022), whereas the Holstein bovine sperms can reach up to 70 μm (Jiang et al., 2019). Based on theoretical predictions, an effective capture of boar sperms would require a capture space of at least 25 μm, while bovine sperms would require approximately 35 μm or more.

To validate this hypothesis, we tested three different capture space sizes—13.8, 23.8, and 70 μm—under identical conditions (square wave, applied frequency of 3 MHz, applied voltage of 20 Vpp, and C1+W7 electrode configuration) and compared the capture efficiency of both boar and bovine sperms. Figure 4e illustrates the capture efficiency of boar sperms under different capture space sizes, while Figure 4f presents the corresponding results for bovine sperms under the same conditions. The experimental results showed that a 23.8 μm capture space was sufficient for effective trapping of boar sperms. However, this space was still inadequate for the larger bovine sperms, whose capture efficiency remained relatively low. When the capture space was increased to 70 μm, the capture efficiency for bovine sperms significantly improved and became comparable to that of boar sperms. These findings further confirm that the size of the capture space must be tailored to the sperm size of each species in order to achieve optimal capture efficiency. Supplementary Movies 1, 2 show representative capture behavior of boar and bovine sperms, respectively. Based on these results, we conclude that the chip design offers flexibility in adjusting electrode spacing according to sperm size across different species. For example, the average length of human sperm is about 60 μm (Sunanda et al., 2018), suggesting that a capture space of at least 30 μm would be required for efficient trapping—well within the adjustable range of our current chip design.

However, the chip has certain limitations in terms of size range. At present, the capture space can only be adjusted between 10 μm and 70 μm. For species with significantly longer sperms—such as rats (160 μm) or honey possum (349 μm) (Thompson et al., 2018)—half their length exceeds the maximum available capture space. As a result, the chip is unable to effectively capture these longer sperm types by capture space adjustment alone. This represents a potential limitation of the current chip design in capturing extremely long sperm samples.

## Conclusion

In this study, we employed a embedded 3D TiN nano-electrode arrays that combines strong electric field generation with high biocompatibility to capture motile sperms. Fabricated using CMOS technology, the electrodes are miniaturized to the nanoscale, encapsulated with an insulating layer, and surrounded by elevated structures. This design enhances electric field strength while maintaining excellent biocompatibility. This innovative dielectrophoretic chip integrates both electrode-based dielectrophoresis and insulator-based dielectrophoresis characteristics, achieving the strong electric field characteristic of electrode-based dielectrophoresis for effective sperm capture, while maintaining the excellent biocompatibility and low Joule heating associated with insulator-based dielectrophoresis. As a result, this chip offers high electric field intensity, low thermal fluctuations, high throughput, and minimal damage to biological samples (i.e., sperm). Throughout the experiments, we evaluated the impact of our chip on sperm viability and the effects of waveform and capture space size on capture efficiency across species (boar and bovine). We first calculated the generated Joule heat and measured the temperature rise before and after dielectrophoretic operation. Even under square wave excitation at applied 20 Vpp for 30 s, the chip’s temperature only increased by 1.7°C—significantly lower than the 3.3°C observed in conventional micro-electrode chips. The calculated total Joule heat was just 1.55 × 10^−5^ J, indicating negligible thermal impact on sperms. This was further supported by viability tests, which showed no significant reduction in sperm survival after capturing, confirming the chip’s high biocompatibility and suitability for a wide range of operating conditions.

In waveform comparison experiments, we observed that square waves improved sperm capture efficiency by approximately 10% compared to sine waves, consistent across both boar and bovine sperms. Additionally, increasing the capture space to exceed half the sperm length significantly enhanced capture efficiency: ≥25 μm for boar sperms and ≥35 μm for bovine sperms. With these adjustments, bovine sperm capture efficiency rose to levels comparable with boar sperms, demonstrating the chip’s adaptability to different species.

The optimal capture condition was achieved using square wave excitation (3 MHz, 20 Vpp) with the C1+W7 electrode combination, resulting in a maximum capture efficiency of 65.54% ± 1.07% for boar sperms and 63.25% for bovine sperms. Although this is lower than the capture efficiency typically reported for immotile cells or bacteria (Fernandez et al., 2017; Zhou et al., 2002), it is noteworthy given the high motility of sperms. Capturing over half of the motile sperm population indicates substantial potential for practical applications. It should be noted, however, that due to size differences among species, the current chip’s maximum capture space of 70 μm limits its suitability to sperms with total lengths of approximately 140 μm or less. In the future, we would aim to adapt the chip for a wider range of species by adjusting the size of the electrode control unit, potentially enabling the establishment of a comprehensive database on sperm capture efficiency, and ultimately contributing to the development of a complete sperm capture system.

In conclusion, this study demonstrates that the embedded 3D TiN nano-electrode arrays, which feature a unique design, can effectively capture motile sperms while offering high tunability, cross-species adaptability, and low thermal interference. These attributes position the chip as a promising platform for future applications in dynamic cell capture, high-throughput screening, and biological analysis.

## Data Availability

The original contributions presented in the study are included in the article/Supplementary Material, further inquiries can be directed to the corresponding author.

## References

[B1] Abd Rahman N. Ibrahim F. Yafouz B. (2017). Dielectrophoresis for biomedical sciences applications: a review. Sensors 17, 449. 10.3390/s17030449 28245552 PMC5375735

[B2] Boldt N. P. Weirauch L. Späth J. M. Kerst U. Birkholz M. Baune M. (2025). When to use rectangular waveforms in dielectrophoresis application to increase separation and sorting efficiency. ELECTROPHORESIS 46 (1-2), 104–111. 10.1002/elps.202400164 39607118 PMC11773296

[B3] Chakraborty S. Saha S. (2022). Understanding sperm motility mechanisms and the implication of sperm surface molecules in promoting motility. Middle East Fertil. Soc. J. 27 (1), 4. 10.1186/s43043-022-00094-7

[B4] Contreras Dávila G. Gómez-Quiñones J. I. Perez-Gonzalez V. H. Martinez-Duarte R. (2016). Assessing the advantages of using square wave signals for particle trapping in carbon-electrode dielectrophoresis. ECS Trans. 72 (1), 105–114. 10.1149/07201.0105ecst

[B5] Dararatana N. Tuantranont A. Wongtawan T. Oonkhanond B. (2015). “The dielectrophoresis microfluidic chip for cell separation: case study of separation of floating cell and moving cells,” in 2015 8th Biomedical Engineering International Conference (BMEiCON), 1–5. 10.1109/bmeicon.2015.7399511

[B6] Fernandez R. E. Rohani A. Farmehini V. Swami N. S. (2017). Review: microbial analysis in dielectrophoretic microfluidic systems. Anal. Chim. Acta 966, 11–33. 10.1016/j.aca.2017.02.024 28372723 PMC5424535

[B7] Jiang H. Kwon J. W. Lee S. Jo Y. J. Namgoong S. Yao X. R. (2019). Reconstruction of bovine spermatozoa substances distribution and morphological differences between holstein and Korean native cattle using three-dimensional refractive index tomography. Sci. Rep. 9 (1), 8774. 10.1038/s41598-019-45174-3 31217533 PMC6584538

[B8] Julius L. A. N. Scheidt H. Krishnan G. Becker M. Nassar O. Torres-Delgado S. M. (2023). Dynamic dielectrophoretic cell manipulation is enabled by an innovative electronics platform. Biosens. Bioelectron. X 14, 100333. 10.1016/j.biosx.2023.100333

[B9] Katigbak R. D. Turchini G. M. de Graaf S. P. Kong L. Dumée L. F. (2019). Review on sperm sorting technologies and sperm properties toward new separation methods via the interface of biochemistry and material science. Adv. Biosyst. 3 (9), 1900079. 10.1002/adbi.201900079 32648656

[B10] Koh J. B. Y. Marcos (2014). Effect of dielectrophoresis on spermatozoa. Microfluid. Nanofluidics 17 (4), 613–622. 10.1007/s10404-014-1342-x

[B11] Li J. Zhao W. Zhu J. Wang S. Ju H. Chen S. (2023). Temperature elevation during semen delivery deteriorates boar sperm quality by promoting apoptosis. Anim. (Basel) 13 (20), 3203. 10.3390/ani13203203 PMC10603671 37893927

[B12] Liao I.-H. Shiau J.-H. Chou K.-R. Lien C.-L. Cheng C.-M. (2025). Innovative CMOS-fabricated dielectrophoretic chip: application of 3D TiN nano-electrode arrays with adjustable electrode spacing in sperm capture. Front. Bioeng. Biotechnol. 13, 1565743–2025. 10.3389/fbioe.2025.1565743 40213642 PMC11983159

[B13] Lien C.-L. Yuan C.-J. (2019). The development of CMOS amperometric sensing chip with a novel 3-Dimensional TiN nano-electrode array. Sensors 19, 994. 10.3390/s19050994 30813577 PMC6427664

[B14] Lu H.-J. Liao I.-H. Lien C.-L. Shiau J.-H. Shen C.-F. Chou K.-R. (2024). Dielectrophoretic capture of *Escherichia coli* and boar sperms using ULSI-fabricated three-dimensional protruding TiN nano-electrode arrays. Front. Bioeng. Biotechnol. 12, 1470606. 10.3389/fbioe.2024.1470606 39411058 PMC11473421

[B15] Morgan H. Hughes M. P. Green N. G. (1999). Separation of submicron bioparticles by dielectrophoresis. Biophysical J. 77 (1), 516–525. 10.1016/s0006-3495(99)76908-0 PMC1300348 10388776

[B16] Mortimer D. (2020). A technical note on the assessment of human sperm vitality using eosin-nigrosin staining. Reprod. Biomed. Online 40 (6), 851–855. 10.1016/j.rbmo.2020.03.002 32362570

[B17] Park J. Komori T. Uda T. Miyajima K. Fujii T. Kim S. H. (2020). Sequential cell-processing system by integrating hydrodynamic purification and dielectrophoretic trapping for analyses of suspended cancer cells. Micromachines 11, 47. 10.3390/mi11010047 PMC7019789 31905986

[B18] Pesch G. R. Du F. (2021). A review of dielectrophoretic separation and classification of non-biological particles. ELECTROPHORESIS 42 (1-2), 134–152. 10.1002/elps.202000137 32667696

[B19] Shuchat S. Park S. Kol S. Yossifon G. (2019). Distinct and independent dielectrophoretic behavior of the head and tail of sperm and its potential for the safe sorting and isolation of rare spermatozoa. ELECTROPHORESIS 40 (11), 1606–1614. 10.1002/elps.201800437 30892707

[B20] Sunanda P. Panda B. Dash C. Padhy R. N. Routray P. (2018). An illustration of human sperm morphology and their functional ability among different group of subfertile males. Andrology 6 (5), 680–689. 10.1111/andr.12500 29959832

[B21] Szablicka D. Wysokińska A. Pawlak A. Roman K. (2022). Morphometry of boar spermatozoa in semen stored at 17 °C-The influence of the staining technique. Anim. (Basel) 12 (15), 1888. 10.3390/ani12151888 PMC9332225 35892538

[B22] Tada S. Seki Y. (2022). Analysis of temperature field in the dielectrophoresis-based microfluidic cell separation device. Fluids 7, 263. 10.3390/fluids7080263

[B23] Thompson S. K. Kutchy N. A. Kwok S. Rosyada Z. N. A. Imumorin I. G. Purwantara B. (2018). Review: sperm: comparative morphology and function related to altered reproductive strategies and fertility in mammals. Prof. Animal Sci. 34 (6), 558–565. 10.15232/pas.2018-01748

[B24] Wongtawan T. Dararatana N. Thongkittidilok C. Kornmatitsuk S. Oonkhanond B. (2020). Enrichment of bovine X-sperm using microfluidic dielectrophoretic chip: a proof-of- concept study. Heliyon 6 (11), e05483. 10.1016/j.heliyon.2020.e05483 33241151 PMC7672294

[B25] Zhang H. Chang H. Neuzil P. (2019). DEP-on-a-Chip: dielectrophoresis applied to microfluidic platforms. Micromachines (Basel) 10 (6), 423. 10.3390/mi10060423 31238556 PMC6630590

[B26] Zhou G. Imamura M. Suehiro J. Hara M. (2002). “A dielectrophoretic filter for separation and collection of fine particles suspended in liquid,” in Conference Record of the 2002 IEEE Industry Applications Conference. 37th IAS Annual Meeting (Cat. No.02CH37344).

