# Supplementary material for: CMOS-fabricated dielectrophoretic chip with embedded 3D TiN nano-electrode arrays for sperm capture and sperm damage reduction
Source: Front Bioeng Biotechnol. 2025 Jul 14;13:1635799. doi: 10.3389/fbioe.2025.1635799 (PMC12301885; doi:10.3389/fbioe.2025.1635799)
Supplement: Supplementary file 3 [file DataSheet1.docx]

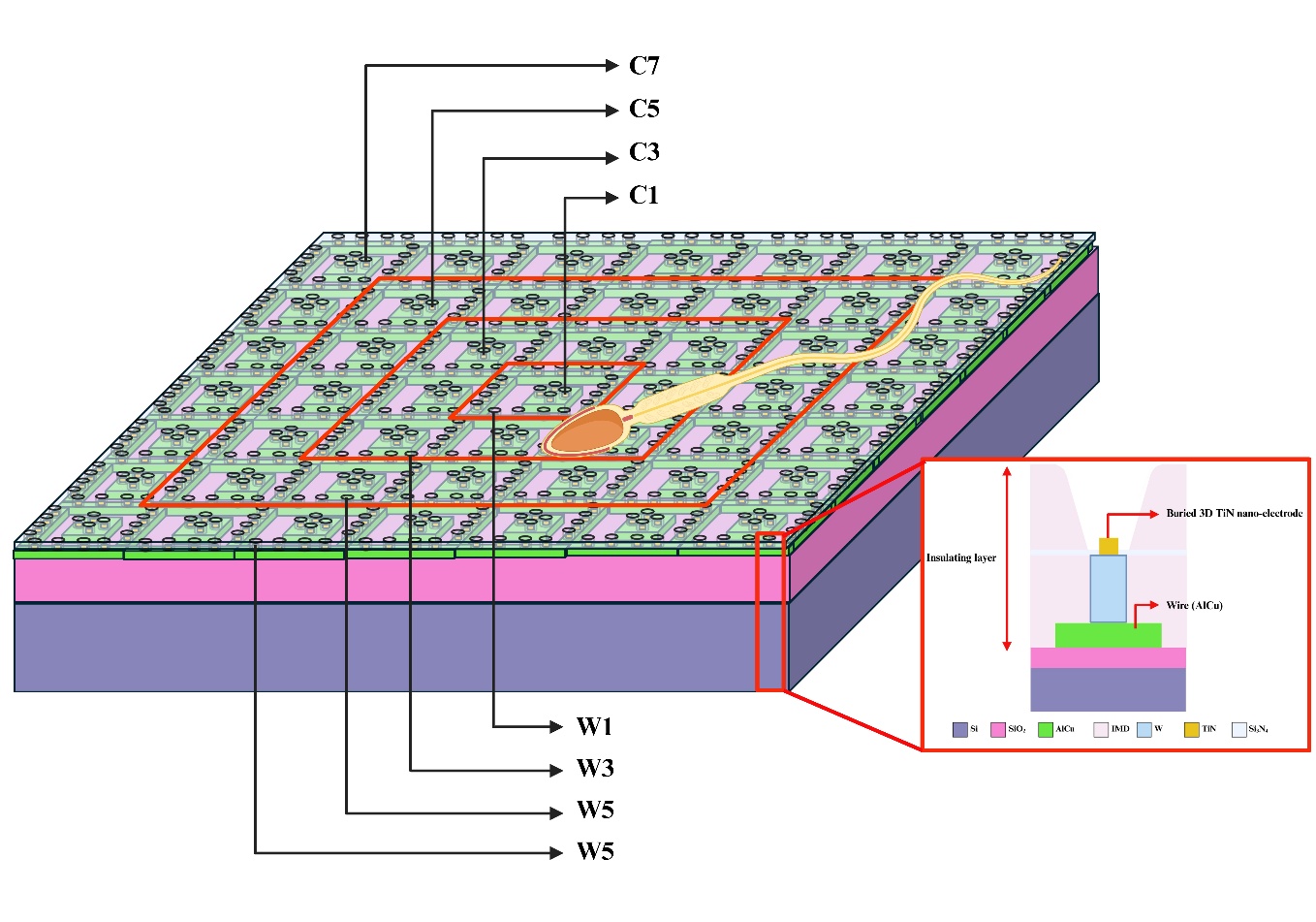


**Figure S1.** Cross-sectional view of the electrode control unit.

**Table S1.** Sperm concentration and conductivity.

|  | Boar sperm (Landrace) | Bovine sperm (Holstein) |
| --- | --- | --- |
| Concentration (sperm/mL) | $1.1\times{10}^{8}$ | $0.11\times{10}^{8}$ |
| Conductivity (mS/cm) | 10.83 | 10.34 |
| Number of sperm observed | 200-300 sperms | 20-40 sperms |
